# The Exaptation of HERV-H: Evolutionary Analyses Reveal the Genomic Features of Highly Transcribed Elements

**DOI:** 10.3389/fimmu.2019.01339

**Published:** 2019-07-09

**Authors:** Patrick Gemmell, Jotun Hein, Aris Katzourakis

**Affiliations:** ^1^Department of Zoology, University of Oxford, Oxford, United Kingdom; ^2^Department of Statistics, University of Oxford, Oxford, United Kingdom

**Keywords:** HERV-H, endogenous retrovirus, transcription, stem cell, exaptation

## Abstract

HERV-H endogenous retroviruses are thought to be essential to stem cell identity in humans. We embrace several decades of HERV-H research in order to relate the transcription of HERV-H loci to their genomic structure. We find that highly transcribed HERV-H loci are younger, more fragmented, and less likely to be present in other primate genomes. We also show that repeats in HERV-H LTRs are correlated to where loci are transcribed: type-I LTRs associate with stem cells while type-II repeats associate with embryonic cells. Our findings are generally in line with what is known about endogenous retrovirus biology but we find that the presence of the zinc finger motif containing region of *gag* is positively correlated with transcription. This leads us to suggest a possible explanation for why an unusually large proportion of HERV-H loci have been preserved in non-solo-LTR form.

## Background

Endogenous retroviruses (ERVs) are the result of germ line retroviral integrations that are passed from one generation to another in a Mendelian fashion. At integration, a typical ERV locus contains the viral genes *gag, pol*, and *env*, as well as two flanking long terminal repeats (LTRs) that are identical. Once present in a population, ERVs may increase in number via reinfection or retrotransposition (1) until all active members of the family are silenced by host defenses (1, 2), degraded by mutations, or truncated by solo-LTR formation (1). Most human endogenous retroviruses (HERVs) are highly degraded, with a small number of described cases where they have been co-opted by their host, preserving their sequence, and now play roles in human biology. HERV derived interferon-inducible enhancers regulate human innate immune pathways (3), with the widespread recruitment of endogenous viruses in host immunity across animals pointing to a systematic evolutionary process (4). Furthermore, the ERV derived *syncytins* are important for placentation (5), and recent studies suggest that members of the HERV-H family are important to the maintenance of stem cell identity (6, 7).

The HERV-H family—formerly known as RTVL-H, for retrovirus like sequence primed with a primer binding site homologous to histidine tRNA—has been studied down to the level of individual insertions on many occasions in the past 30 years. The LTRs of HERV-H promote its transcription, and variation in these regions have been used to divide the family into three subtypes: type-I, type-Ia, and type-II (8, 9). The recombinant type-Ia subtype was originally thought to have expanded recently and to have stronger transcription than loci with pure type-I or type-II LTRs (9); however, the relative youth of type-Ia repeats was later brought into question by Anderssen et al. (10), who discovered type-Ia repeats in the marmoset, a New World monkey species. Thus, considerable effort has been made to identify the structural variations of HERV-H that are important to its biological activity.

The sequencing of the human genome was important to a more comprehensive analysis of HERV-H. A study of the majority of full-length HERV-H integrations was performed by Jern et al. (11), who clustered HERV-H into two groups, the larger HERV-H group of 926 full-length elements and the smaller HERV-H like group of 92 full-length elements. Within the larger HERV-H group, Jern et al. (11) used previously studied variants of HERV-H (12, 13) to define two further subgroups: an older group of 77 RGH2-like elements (13) having a fairly intact *pol* and more frequently containing *env*; and a younger group of 705 RTVLH2-like elements (12) having more *pol* deletions and less frequently containing *env*. The 926 bona-fide HERV-H elements in the human genome were subsequently studied in some detail resulting in an annotated consensus sequence (14). In an unrelated study on ERV replication mechanisms, a small number of relatively intact HERV-H (presumably the RGH2-like elements) were found to have low *env* dN/dS. It was suggested that these elements had replicated via re-infection and were also responsible for copying other less intact members of the HERV-H family (15). These studies provided further important information on the origin and diversity of HERV-H within the human genome.

In the last few years interest in HERV-H has redoubled as the relationship between retrotransposons and stem cells has become a focus of research. In the case of HERV-H, there is evidence that HERV-H transcripts are upregulated in, and necessary for, the maintenance of human stem cell identity. This evidence derives from studies including those of Wang et al. (7) who show that half of the full-length HERV-H in the human genome are bound by pluripotency associated transcription factors NANOG, OCT5, and LBP9. These loci produce chimeric hESC (human embryonic stem cell) and hiPSC (human induced pluripotent stem cell) specific transcripts and long non-coding RNAs. By disrupting HERV-H or LBP9, Wang et al. (7) could demonstrate that some HERV-H elements play an essential biological role: differential markers were upregulated while pluripotency-associated transcription factors were downregulated, so that an ability for self-renewal was shown to be impaired. The same year, Lu et al. (6) reached a similar conclusion on the importance of HERV-H after discovering that interfering with HERV-H transcripts in hESCs led to modified cells becoming more fibroblast like, with concomitant changes in the appropriate transcriptional markers. More recently, Gemmell et al. (16) showed that the most highly transcribed HERV-H have diverged quickly, and are presumably under directional selection, while Göke et al. (17) studied HERV-H transcription in different cell types and concluded that HERV-H loci are stage-specific regulatory elements in humans. Combined, these studies suggest that HERV-H sequences have been exapted by their hosts, whereby retrovirally derived sequences have been functionally co-opted for their roles in host biology. These exciting recent results have yet to be integrated with the tradition of studying HERV-H from an evolutionary perspective.

**Figure 1:**
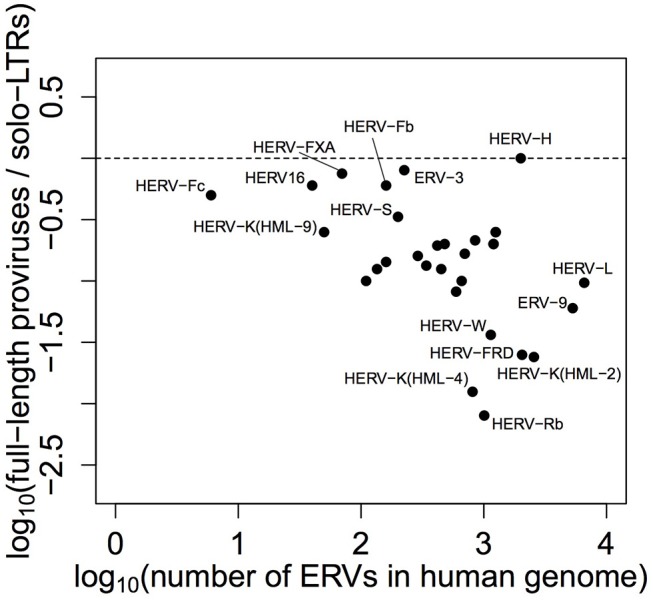
Ratio of ERVs found in full-length form to solo-LTR form, plotted against total number of ERVs, by family—in humans, HERV-H has the highest full-length provirus to solo-LTR ratio (1:1) and is particularly unusual among larger retroviral families. The data plotted in this figure were originally presented by Bannert and Kurth (18) in tabular form.

Given the current interest in HERV-H, we think it important to capitalize on several decades of detailed work on the family, including the aforementioned consensus and the LTR repeat types that have previously been shown to affect transcription. Additionally, we think it notable that an especially large number of HERV-H loci have been maintained in non-solo-LTR form (see Figure 1), an oddity that has been raised but not resolved in the recent literature (19–21). For these reasons, the crux of this study is as follows: what is the relationship between the genomic characteristics of a HERV-H locus and the level at which it is transcribed? Below we identify the features of HERV-H that are significantly correlated with transcription and show how age, integrity, and the LTR repeat type of a locus affect its transcription levels. We also demonstrate that there is a relationship between *gag* and transcription, and conjecture that the presence of zinc finger motifs in the terminal region of this gene might be related to why so many HERV-H loci are maintained in a full-length state.

## Results

**Figure 2:**
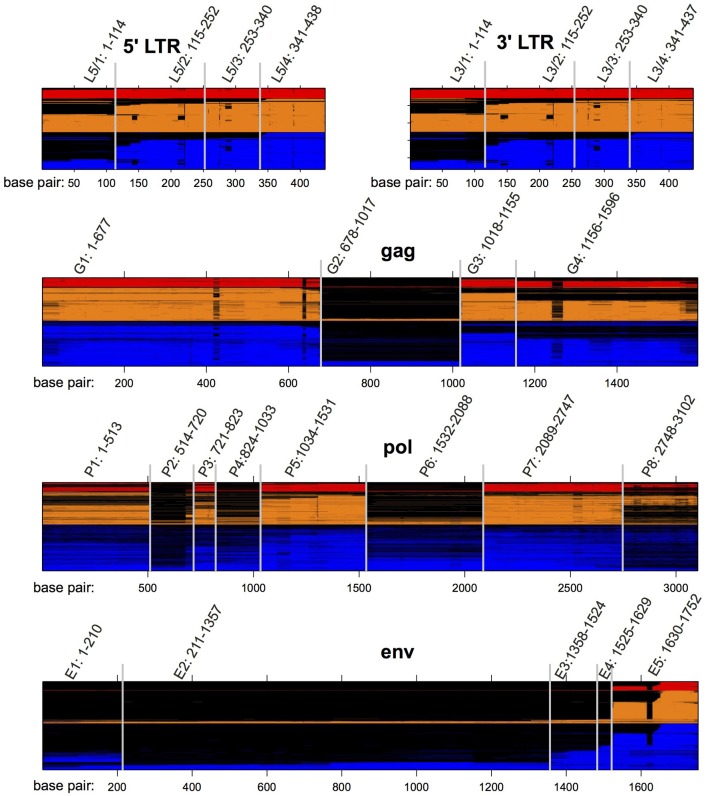
HERV-H sequences from the human genome aligned to the published consensus of Jern et al. (14). Loci are colored according to the categorical transcription levels of Wang et al. (7): red (highly active); orange (moderately active); blue (inactive). Gaps in the alignment are colored black. The locations of regions L5/1–L5/4, G1–G4, P1–P8, E1–E5, and L3/1–3/4 are also noted.

We produced multiple sequence alignments of the HERV-H genes *gag* (1,080 sequences), *pol* (1,126 sequences), and *env* (1,081 sequences), using the consensus HERV-H (14) as a guide. (Complete procedures are provided in the Methods section). We used the same consensus to form alignments of the 5′ (908 sequences) and 3′ (932 sequences) LTRs of HERV-H. Examining the alignments we identified regions that were shared deletions across subsets of viral sequences. We refer to these regions via enumeration from 5′ to 3′ and they are depicted in Figure 2.

BLASTn was used to search for the previously characterized type-I and type-II repeats within the HERV-H LTRs. To place the HERV-H loci in context we obtained their distance from the nearest gene, and also located 847 of the loci in a six-way alignment of primate genomes, thereby gaining some perspective on their structural state in other primates. Finally, we were able to determine the genetic distance (K80 substitution model) between the paired LTRs of 627 of the HERV-H loci—as the divergence between paired LTRs accumulates at a neutral rate, both genetic distance and orthology contribute information about the age of the loci under investigation.

Because HERV-H loci are related, they do not represent independent data points for statistical analysis. To quantify the relationship between HERV-H transcription data (7) and the aforementioned per locus genomic features of HERV-H, we controlled for the non-independence between measurements due to shared evolutionary history by incorporating phylogenetic information. A maximum likelihood (ML) phylogeny of the genes of the HERV-H loci was constructed, and we used a phylogenetic generalized least squares (PGLS) method to control for the non-independence of measurements across the HERV-H family. Of the 834 loci in the phylogeny, 409 had a complete collection of genomic features (i.e., no missing values) and could therefore be used in our PGLS analysis (Tables 1, 2). Akaike information criterion (AIC) based model selection and plots were used to explore model space and we formulated minimum adequate models to explain the features of our dataset i.e., we carefully explored regression space and include here models containing statistically informative explanatory variables. We provide our data in Additional File 1. The response variable in our analyses was the logarithm of per locus mean transcription in reads per kilobase per million reads (RPKM) and PGLS regressions were performed using measurements from three distinct cell types defined by Wang et al. (7): somatic (32 sample types); embryonic (114 sample types); and stem (55 hESC and 25 hiPSC sample types). An overview of the methods of Wang et al. is included in our own methods section, which also describes how to link our data to theirs.

**Table 1:** PGLS applied to transcription data from embryonic cells for 409 HERV-H loci: λ = 0.48 (0.17, 0.78); *R*^2^ = 0.24.

**Coefficient**	**Estimate**	**2.5%**	**97.5%**	**Std. err**.	***t* value**	***p*-value**
(Intercept)	−5.26	−6.92	−3.60	0.85	−6.22	**p** < 0.01
L5/1	0.76	0.23	1.29	0.27	2.82	**p** < 0.01
G4	0.46	0.10	0.82	0.18	2.50	**p** = 0.01
P6	−0.98	−1.72	−0.23	0.38	−2.57	**p** = 0.01
Type-II	2.24	1.32	3.16	0.47	4.76	**p** < 0.01
Type-Ia	0.71	−0.55	1.97	0.64	1.11	**p** = 0.27

**Table 2:** PGLS applied to transcription data from stem cells for 409 HERV-H loci: λ = 0.50 (0.27, 0.73); *R*^2^ = 0.13.

**Coefficient**	**Estimate**	**2.5%**	**97.5%**	**Std. err**.	***t* value**	***p*-value**
(Intercept)	−6.23	−8.17	−4.29	0.99	−6.30	**p** < 0.01
L5/1	1.28	0.72	1.85	0.29	4.46	**p** < 0.01
G4	0.75	0.21	1.29	0.27	2.72	**p** = 0.01
P6	−0.91	−1.75	−0.06	0.43	−2.11	**p** = 0.04
LTR divergence	−21.21	−32.54	−9.89	5.78	−3.67	**p** < 0.01
Type-I	0.88	0.44	1.33	0.23	3.87	**p** < 0.01

### Qualitative Analysis

In this section we discuss what we notice about our results by looking at them. This motivates the quantitative analysis that follows in the next section.

**Figure 3:**
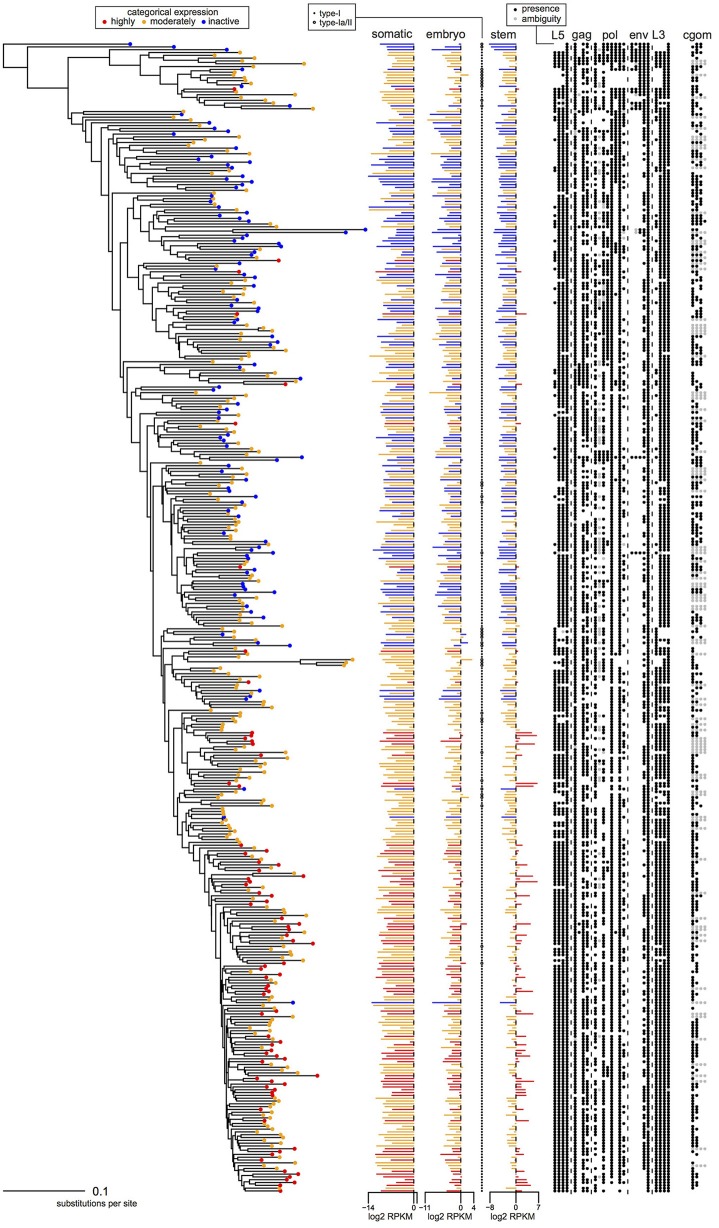
Phylogeny, transcription levels, and per locus genomic features of the 409 HERV-H loci from the human genome that were analyzed using PGLS. Loci are colored according to the categorical transcription levels of Wang et al. (7): highly active (red); moderately active (orange); inactive (blue). Columns *L5, gag, pol, env*, and *L3* indicate the presence/absence of regions L5/1–L5/4, G1–G4, P1–P8, E1–E5, and L3/1–L3/4, respectively; column *cgom* indicates the substantive presence/absence of each locus in chimpanzee, gorilla, orangutan, and macaque.

**Figure 4:**
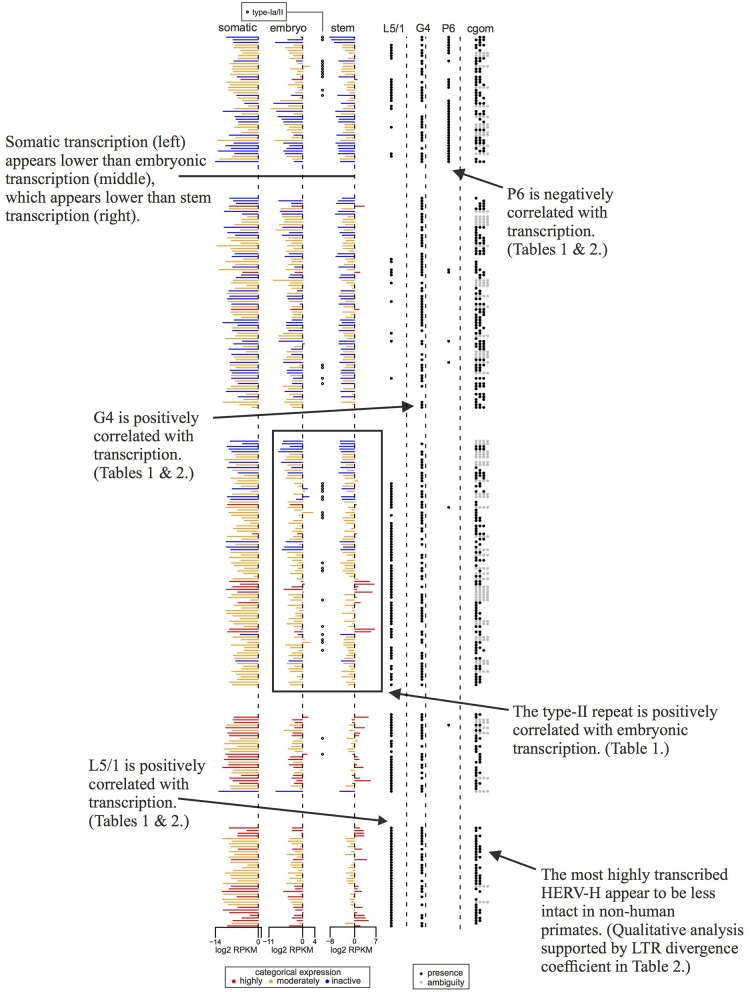
Concise guide to Figure 3 showing transcription levels and per locus genomic features of a subset of the 409 HERV-H loci. Rows are ordered, colored, and sourced as per Figure 3, though some rows are omitted to aid clarity. Annotations highlight aspects of our results that are discussed in detail in the main article text.

The relationship between HERV-H phylogeny, transcription, and genomic structure is visually summarized in Figure 3. To aid the reader, a simplified and annotated partial version of this figure is also provided in Figure 4. The tips of the tree in Figure 3 are colored according to the categorical transcription level assigned to them by Wang et al. (7) (see Methods). Upon visual inspection, it can be seen that loci annotated as highly transcribed by Wang et al. (7) are located toward the bottom of the ladderized tree, and we confirmed that this phenomenon was also true of phylogenies built using LTRs and from amino-acid or nucleotide trees of individual HERV-H genes (some examples are contained in Additional File 2). Thus, HERV-H transcription appears to have a phylogenetic component.

In which cells is HERV-H active? Figures 3, 4 represent the average transcription of HERV-H loci across cells categorized as somatic, embryonic, or stem. The figure shows a hierarchy of HERV-H transcription such that transcription is typically lowest in somatic cells, and higher in embryonic cells and stem cells in turn. Note that this comparison between different cell types involves contrasting data gathered in different ways (e.g., cell culture vs. primary sources) by different labs and that any systematic effect this leads to is not something that we correct for.

LTRs identified as type-I outnumber those identified as type-Ia/II. However, considering transcription in conjunction with LTR subtypes, one can also see that type-Ia/II loci are distributed throughout the tree and that type-II repeats are associated with higher levels of embryonic transcription, even if a locus is categorically characterized as inactive or moderately active by Wang et al. (7). Therefore, in agreement with Göke et al. (17), we seem to identify stage-specific transcription, though in this case we relate it to particular well-studied repeat types [see e.g., (10)].

If transcription is positively correlated with function, our results suggest that many parts of consensus HERV-H might have deleterious effects that prevent their exaptation. Columns *L5, gag, pol, env*, and *L3* of Figure 3 indicate the presence or absence of the viral regions L5/1–L5/4, G1–G4, P1–P8, E1–E5, and L3/1–3/4 at particular loci. Highly active loci toward the bottom of the tree can be seen to have highly intact LTRs, while some less transcribed loci toward the top of the tree have less intact LTRs. Less active older loci toward the top of the tree tend to have relatively complete internal regions when compared to the active loci at the bottom of the figure.

The status of active HERV-H in other primates is also of interest. The sequences used to build the phylogeny in Figure 3 come from the human genome. A rough indication of the age and status of HERV-H loci in other primates is given by the column *cgom*. Loci present in chimpanzee, gorilla, orangutan, or macaque at a minimum of 25% of the level that they appear in human, and that therefore presumably consist of more than a solo-LTR, are marked with a dot as appropriate. Visual inspection of Figures 3, 4 shows that the less derived and less active HERV-H loci that are located toward the top of the tree appear more often present in a substantive way in other primates; the active and derived loci toward the bottom of the tree appear to be absent or degraded in distant primates. This suggests that if HERV-H is important to stem cell biology in non-human primates, then the loci that are involved might be different to those that are involved in humans.

### Statistical Results

The above observations—younger loci are more transcribed; loci with less intact genes are more transcribed; loci with more intact LTRs are more transcribed; the repeat type of an LTR is relevant to where the corresponding HERV-H is transcribed—are supported by a statistical analysis and robust to phylogenetic non-independence. Two PGLS regressions are reported in detail: Table 1 displays a regression for transcription measurements from embryonic cells and Table 2 shows a similar analysis of stem cell data. Both regressions were constructed with respect to the same 409 loci though each model contains only the explanatory variables that were found to be important from a statistical perspective.

In embryonic cells, the presence of region P6 of *pol* is negatively correlated with transcription. The P6 region of *pol* has previously been identified with an RNaseH motif and the 5′ end of the integrase domain (14). Positive correlations between embryonic transcription and the presence of the LTR region L5/1, as well as between embryonic transcription and type-II repeats, are also seen. For stem cells, the situation is similar, in that P6 is negatively correlated with transcription, whereas L5/1 is positively correlated with transcription. However, Table 2 also shows that in stem cells it is type-I LTRs (indicator TI + UI) that are positively correlated with transcription, and that the older an ERV is the less active it is likely to be. Surprisingly, given that Figures 3, 4 suggest that intact loci tend to be less transcribed, the presence of the G4 region of *gag* is positively correlated with transcription in both embryonic cells and stem cells.

A coefficient of determination corrected to take account of correlation structure [see (22), p. 224] can be applied to the regressions presented in Tables 1, 2. The regression describing embryonic data has *R*^2^ = 0.24, and therefore explains nearly a quarter of the variance in the transcription of HERV-H using only a few features of the loci themselves. The model describing stem cell data has *R*^2^ = 0.13, and seems to underfit the most highly transcribed HERV-H loci. Both PGLS models possess intermediate levels of phylogenetic signal which suggests that the evolutionary relationship between HERV-H loci must be taken into account in order to make correct statistical inferences relating structure and transcription. As expected, given that there is no reported role for HERV-H in somatic cells, we were unable to produce a PGLS regression with *R*^2^ > 0.01 for somatic cell measurements—therefore we do not think there is a meaningful relationship between the structure of the majority of HERV-H loci and their somatic transcription.

## Discussion and Conclusions

We found the repeat class of LTRs to be related to where the corresponding HERV-H is transcribed. HERV-H loci classified as type-I were roughly twice as highly transcribed in stem cells when compared to other loci. On the other hand, we found the presence of a type-II repeat increased transcription of HERV-H loci by a factor of four in embryonic cells. The finding that type-II repeats are preferentially transcribed in embryonic cells is potentially important in light of the widespread interest in cultivating naive human cells. Mouse ESC cultures have been shown to contain sub-populations of naive-like cells that have totipotent properties (23), and these totipotent cells have transcriptional characteristics that are usually associated with 2-cell embryos. Our analyses show that the type-II repeat is correlated with HERV-H transcription in early human embryonic cells. At the same time, these loci with type-II repeats do not appear to be a focus of Wang et al. (7) as they do not usually cluster into the highly active category (Figures 3, 4). As the work of Macfarlan et al. (23) shows that the natural timing of the transcription of repeat loci is relevant for identifying naive-like cells in mouse, it is worth considering whether a similar situation is true for humans also.

We also demonstrated that the integrity of HERV-H LTRs is important to the magnitude of HERV-H transcription. In particular, transcription of HERV-H is consistently correlated with the presence of the first 114 bp (L5/1) of the consensus LTR. This short sequence is part of the larger U3 region and is known to contain a MYB binding site (76–80) as well as to finish with an Sp1 binding site (105–114). The correlation makes sense given Sp1 binding sites have been shown to be important in previous assays (10, 24).

A related feature of our results is the fragmented nature of the *pol* and *env* genes of many HERV-H loci (Figures 2, 3). It has previously been shown that the decay of *env* is a common feature of the most prolific families of endogenous retroviruses (25) and HERV-H seems to be typical in this respect. In addition it is sensible to assume that a more complete (i.e., longer) *pol* or *env* will contain more sequence that triggers host defenses (2) or is more likely to be involved in harmful ectopic recombination events [e.g., (26)]. In particular, we find here that the P6 region of *pol* is negatively correlated with transcription to the extent that the presence of P6 is associated with a roughly 2-fold decrease in HERV-H transcription. It will be interesting to determine whether the P6 region attracts repressive chromatin/DNA marks that could explain this observation. This result shows that, even today, more complete HERV-H loci are subject to reduced transcription in their host.

In light of the preceding discussion it is striking that the *gag* gene is relatively complete in the majority of HERV-H, and that the presence of the G4 region of *gag* is associated with a 38 and 68% increase in HERV-H transcription in embryonic and stem cells, respectively. The consensus HERV-H *gag* has previously been annotated and was described as containing two zinc finger motifs (14). These motifs are located in region G4 (positions 1390–1431 and 1459–1497) and would have originally enabled proteins encoded by the exogenous progenitor of HERV-H to bind single-stranded viral RNA as part of a packaging process. The association between these motifs and HERV-H transcription levels may be a statistical coincidence, but it is unexpected enough to encourage speculation on the alternative that there might be a causal explanation for the relationship.

We draw upon recent research that shows that endogenous viral sequences have been frequently repurposed by their hosts to provide functional roles in host immunity. This process results in virally derived genes that are now functioning as host immune genes, which have been termed endogenous viral element-derived immunity (EDIs) (27). We propose that just as exogenous zinc finger motifs enable retroviral proteins to package RNA to the benefit of an exogenous virus, so too might endogenous zinc finger motifs have at one time enabled HERV-H proteins to bind viral RNA to the benefit of the host. The general phenomenon of viral material being captured by a host for immune benefits is well-established and occurs via many mechanisms and in many organisms (27). In this case our suggestion is that insofar as zinc finger motif-complete products of HERV-H were in other ways defective, or bound related viruses that they did not package, they would act in competition with genuinely effective packaging proteins and therefore as antagonists against viral reinfection. This scenario invokes a new role for zinc finger domains in host defense as compared to their (unrelated) known role as part of host KRAB zinc finger based defenses (28–31).

Reflecting on the above discussion we conclude with the following narrative. In the distant past exogenous HERV-H was a complete retrovirus adapted to horizontal transmission and at some point roughly 35 million years ago an endogenization occurred (32). It is possible that HERV-H then drifted to fixation. The intact nature of HERV-H *gag* is in stark contrast to the fragmented *pol* and *env* genes that are the hallmarks of prolific endogenous retroviruses. If we suppose the relationship between the zinc finger motif containing region of *gag* and transcription is important, this would go some way toward explaining why HERV-H is present at a roughly 1:1 full-length to solo-LTR ratio in the human genome. This ratio is extraordinary, but it could be that while HERV-H LTRs drive transcription, at some point in time the zinc finger motifs in the last third of *gag* made some HERV-H loci useful to the host, and consequently more likely to be at first tolerated and then later co-opted for a role in stem cell identity. This would suggest we view co-opted HERV-H as a biologically convenient aggregation of LTRs and the G4 region of *gag*, both embedded in an ancestral ERV packaging, the remainder of which was of little functional consequence over evolutionary time.

## Methods

### Multiple Sequence Alignments of HERV-H Loci

We obtained the genomic sequence underlying the 1,225 full-length HERV-H loci described by (7) from the UCSC Genome Browser database available at http://genome.ucsc.edu (33), and used Genome Reference Consortium Human Build 37 (GRCh37, h19) for the human genome. We also obtained the corresponding RepeatMasker (http://www.repeatmasker.org) gene annotations from the same database. For the non-human primates, we used the following assembly versions: Chimp: CHIMP2.1.4, Gorilla: gorGor3.1, Orangutan: PPYG2, Macaque: MMUL1.0, Marmoset: C_jacchus3.2.1.

Before aligning the HERV-H loci we first used RepeatMasker annotations to identify any non-HERV-H repeats within the underlying sequences. Such non-HERV-H repeats, for example SINEs or LINEs, would, if ignored, introduce confounding indels into our analysis. For this reason all but the outermost 20 bp of these repeats were removed from the 1,225 HERV-H sequences before constructing alignments. The remaining 40 bp or less of non-HERV-H repeats were flagged so that we could remove them by hand, thereby ensuring that we did not remove mistakenly RepeatMasked genuine HERV-H sequence. Non-HERV-H repeats were found to be rare, so that 80% of the sequence underlying the 1,225 loci remained completely unmodified. The remaining 20% of loci had a median of 12% of their underlying sequence removed.

To identify the *gag, pol*, and *env* genes within the 1,225 HERV-H sequences we used tBLASTn (34). We searched each of the 1,225 loci using a previously published (14) HERV-H consensus sequence as a query. Hits of at least 25 bp in length and with an expect value no more than 10^−6^ were merged in a way that maintained fragment order and sense. The result of this reconstruction was 1,080 *gag*, 1,126 *pol*, and 1,081 *env* genes.

Only 20 of the 1,225 HERV-H loci were not matched by one of the three tBLASTn searches. An inspection using Dfam (35) revealed that these loci were sequences with short internal regions, that contained other retroviral insertions, or that were short overall (one full-length region was only 44 bp).

A similar search and merge process was performed for the 5′ and 3′ LTRs of each of the 1,225 HERV-H loci. In this case the search was performed using BLASTn (34) and resulted in the reconstruction of 908 5′ LTRs and 932 3′ LTRs.

To construct multiple sequence alignments of the genes and LTRs of the HERV-H loci we first pairwise aligned the reconstructed sequences to the appropriate part of the consensus. We then progressively combined these alignments to create five multiple sequence alignments, one for each of the three genes and one for each LTR. Pairwise alignment was conducted with Stretcher (36) and progressive multiple alignment was conducted using MUSCLE (37).

### Identifying Regions Containing Common Deletions

Examining the sequence in each alignment we identified regions that demarcated deletions common to subsets of viral sequences. The presence of sequence in these regions was bimodally distributed and is represented in Figure 2, where the regions are named via enumeration from 5′ to 3′: we identified eight regions in the alignment of HERV-H LTRs (L5/1–L5/4 for the four regions in the 5′ LTR and L3/1–L3/4 for the four regions in the 3′ LTR); four regions in *gag* (G1–G4); eight regions in *pol* (P1–P8); and four regions in *env* (E1–E4). As per Belshaw et al. (15), if <5% of a region was aligned for a particular HERV-H the region was marked as absent at that locus, while if more than 55% of a region was aligned it was marked as present. Ambiguous regions were coded as missing values. The 25 regions we defined were included as indicator variables (i.e., 1 or 0) when constructing statistical models (below).

### Distance to Nearest Gene

The RefSeq gene annotation track was downloaded from the appropriate UCSC Genome Browser database (as detailed above). The distance between the centroid of each HERV-H locus and its nearest neighboring gene was calculated and recorded.

### Characterization of LTR Subtypes

Several examples of HERV-H LTR subtypes (9, 32) are provided by Anderssen et al. (10). We constructed consensus type-I (TI) and type-II (TII) repeats as well as consensus unique-I (UI) and unique-II (UII) sequences based on Figure 2 of the study by Anderssen et al. (10). These consensus sequences were used as queries in a BLASTn search against the 1,225 HERV-H loci. Hits with expect values of no more than 10^−6^ were treated as indicating the presence of the appropriate sequence. Data on the presence of LTR subtype sequences at HERV-H loci are recorded in Additional File 1.

### Pairing Loci to EPO Multiple Alignments

To examine the status of the 1,225 HERV-H full-length loci in other primates we obtained the Enredo-Pecan-Ortheus (EPO) genome scale multiple sequence alignment from Ensembl Release 71 (38). We then used BLASTn to locate the HERV-H loci in the human row of the EPO alignments. Of the 1,225 HERV-H loci, 847 were unambiguously located (unique and exact matches) within a six-way EPO alignment. The remaining loci were not present in the alignments in an unambiguous form. This could be because the region of the human genome they are located in is not included in the EPO alignments, or because the region they are located in was identified as having been duplicated in a primate other than human.

Some uncertainty remains over loci that were unambiguously located. This is because the EPO methodology could fail to identify genuine orthologs and paralogues in the non-human primates. We note that orthology information from the EPO alignments contributes qualitatively to our results but was not used in our statistical analyses.

### Tree Building and Phylogenetic Regression

A supermatrix concatenation of *gag, pol*, and *env* alignments was produced. The alignment was edited by hand to remove short or badly aligned regions and sequences that could not reasonably be assumed to be homologous. The tree building software RAxML 8.2.3 (39) was used to produce a maximum likelihood (ML) tree relating the 834 sequences in the supermatrix alignment. Tree inference was performed under the GTR + γ substitution model.

The supermatrix based tree was rooted by constructing an auxiliary phylogeny of the reverse-transcriptase (RT) region of *pol* (nucleotides 82–576 of the *pol* consensus). The 569 HERV-H sequences with relatively complete RT, having over 140 of 165 possible codons, were first translated and then combined with a panel of 15 HERV-W RTs. An ML tree was constructed from the resulting alignment using the PROTGAMMAAUTO option.

All regression analysis was conducted with the R system (40). Regressions taking into account phylogeny were performed using a phylogenetic generalized least squares (PGLS) approach as introduced by (41). The λ method (42) was used to assess phylogenetic signal. Analyses were conducted using the R package APE (43).

### Transcription Data

Wang et al. (7) previously curated HERV-H transcription data from a large panel of RNA sequencing experiments performed on a variety of cell types. We have subsequently related this data to genomic features in our own study.

In overview, Wang et al. (7) compared uniquely mapped HERV-H reads to other uniquely mapped reads and RPKM (reads per kilobase million) normalized. Wang et al. (7) also used hierarchical clustering to apply one of three labels (highly active, moderately active, or inactive) to each HERV-H locus as a way of summarizing the transcription of a locus across many cell types and experiments. These data are fully available in Supplementary Table 7 of Wang et al. (7) while the provenance of the reads themselves is available in Supplementary Table 4 of the same article. Our data can be related in full to the data of Wang et al. (7) via the first column of our Additional File 1: our serial number 1 to 1,225 corresponds to what Wang et al. refer to as Repeat_ID, which takes values HERVH_1 to HERVH_1225.

The data used by Wang et al. (7) come from a variety of cell types which they grouped into five broad categories including somatic tissue (both tissue specific and mixture), embryonic cells, hESC (human embryonic stem cells), and hiPSC (induced pluripotent stem cells). This processing and grouping of RNA transcription experiment data by Wang et al. (7) was convenient for our study and we relied on their data without subsequent relabeling or adjustment.

## Author Contributions

PG and AK conceived the study, interpreted the results, and participated in the editing of the manuscript. PG performed the analysis. PG was supervised by AK and JH. All authors read and approved the final manuscript.

### Conflict of Interest Statement

The authors declare that the research was conducted in the absence of any commercial or financial relationships that could be construed as a potential conflict of interest.

## Supplementary Material

Additional File 1 This file contains information on the structure of primate HERV-H loci in a machine readable tabular form.

Additional File 2 This file contains trees built using HERV-H LTRs and individual HERV-H genes.
